# The implementation of Xpert MTB/RIF assay for diagnosis of tuberculosis in Nepal: A mixed-methods analysis

**DOI:** 10.1371/journal.pone.0201731

**Published:** 2018-08-10

**Authors:** Basant Joshi, Trisasi Lestari, Stephen Michael Graham, Sushil Chandra Baral, Sharat Chandra Verma, Gokarna Ghimire, Bandana Bhatta, Shyam Prakash Dumre, Adi Utarini

**Affiliations:** 1 Graduate Program in Implementation Research, Public Health, Faculty of Medicine, Public Health and Nursing, Universitas Gadjah Mada, Yogyakarta, Indonesia; 2 Department of Health Policy and Management, Faculty of Medicine, Public Health and Nursing, Universitas Gadjah Mada, Yogyakarta, Indonesia; 3 University of Melbourne Department of Paediatrics and Murdoch Children’s Research Institute, Royal Children’s Hospital, Melbourne, Australia; 4 International Union Against Tuberculosis and Lung Disease, Paris, France; 5 Health Research and Social Development Forum (HERD), Kathmandu, Nepal; 6 National Tuberculosis Centre, Ministry of Health, Bhaktapur, Nepal; 7 Save the Children International, Dhangadhi, Nepal; 8 Institute of Tropical Medicine, Nagasaki University, Nagasaki, Japan; Indian Institute of Technology Delhi, INDIA

## Abstract

**Background:**

Tuberculosis (TB) is a major public health problem in low and middle-income countries. Early detection and enrolment of TB cases is a challenge for National TB Programs.

**Objective:**

To understand the performance and feasibility for scale-up of Xpert MTB/RIF assay for the TB diagnosis in Nepal.

**Design:**

Implementation research employed mixed-method sequential explanatory design. The results of Xpert MTB/RIF assay were analysed in 26 TB diagnostic centres where Xpert machines had been installed before 2015. In-depth interviews and focus group discussions were conducted with stakeholders, purposively selected to represent experiences in centres that were functioning well, poorly or not functioning.

**Results:**

During a one-year period in 2015/16, 23,075 Xpert MTB/RIF assays were performed in 21 diagnostic centres with 22,288 people also tested by sputum microscopy. Among these, 77% had concordant (positive or negative) results, demonstrating fair agreement (Kappa score, 0.3) between test results. Test failure and positivity rates in diagnostic centres ranged from 2.6% to 13.4% and 6.5% to 49%, respectively. The number of cartridges per positive result varied from 2.3 to 10.2. Xpert assay was positive in 3314 (15% of all cases) sputum smear microscopy negative cases. Of 4280 bacteriologically confirmed cases by Xpert assay, 355 (8%) were rifampicin resistant. Xpert machines were no longer functioning regularly throughout the year in 5 diagnostic centres. The main barriers for effective implementation of Xpert in Nepal were the lack of: timely supply of cartridges; replacement of damaged modules; maintenance of Xpert machines; and stock verification for timely procurement of cartridges. Inadequate laboratory infrastructure for maintaining functional Xpert equipment further challenges implementation and scale-up.

**Conclusion:**

The implementation of Xpert MTB/RIF assay has increased case-finding of TB and MDR-TB in Nepal. However, there is a need to improve laboratory performance and strengthen laboratory infrastructure for optimal utilisation and scale-up of Xpert.

## Introduction

Tuberculosis (TB) is the major infectious cause of morbidity and mortality in the world [1]. A recent estimate shows that at least one-quarter of the world's population is infected with *Mycobacterium tuberculosis* [2]. There is a 10% risk of developing TB disease in people infected with TB bacilli, but the risk is higher in young children (<5 years) or immunocompromised persons such as people living with Human Immunodeficiency Virus (HIV). According to the recent WHO Global TB report, there were 10.4 million TB cases globally and 1.67 million people dying from TB in 2016 [3]. Although 26% of the global population lives in the South East Asia (SEA) Region of WHO, the region accounts for 41% of the global burden of TB [4].

In Nepal, nearly half of the population is infected with *M*. *tuberculosis* [5]. According to the Nepal’s National TB Program (NTP) report for 2016 [5], of 32,056 cases registered with NTP in 2015/16, 73% were pulmonary TB (PTB) cases and 75% of these were bacteriologically confirmed. TB in children (≤14 years) accounted for 6% of the total TB caseload registered. It is estimated that in Nepal each year, approximately 166 per 100,000 population develop TB including 20,000 new sputum smear-positive cases, there are 8,000–10,000 cases not diagnosed, and there are 5,000–7,000 deaths due to TB [5].

Early diagnosis of TB and prompt initiation of appropriate treatment to minimize transmission and to reduce TB-related morbidity and mortality are major challenges for TB control programs [6–8]. While intensified case-finding approaches contribute to increased case detection [9–11], novel diagnostic methods can potentially further reduce transmission and mortality due to TB by early diagnosis and effective treatment[12]. The X-pert® MTB/RIF assay (Cepheid Inc, Sunnyvale, CA, USA) is a rapid fully automated real-time DNA-based molecular diagnostic technique [13] with the following advantages: high sensitivity and specificity, determines rifampicin resistance as an indicator for multidrug-resistant (MDR) TB, and can provide a result within two hours [14]. In Nepal, Xpert MTB/RIF was first implemented in 2011/12 for rapid diagnosis of TB in specific populations, as recommended by WHO: children (<15 years); people living with HIV (PLHIV); severe forms of TB; and in presumptive MDR TB cases. Xpert MTB/RIF was available in 26 health facilities by 2015 [5].

There has not been an evaluation of the implementation of Xpert MTB/RIF assay in Nepal since it was introduced. Evaluation is important in order to determine the feasibility and effectiveness of delivery, as well as to assess challenges for sustainability and further expansion [15–17]. Therefore, we evaluated the impact of Xpert MTB/RIF on bacteriological confirmation of TB in Nepal and explored barriers and enablers for effective implementation and future scale up.

## Methods

### Study setting

**Fig 1:**
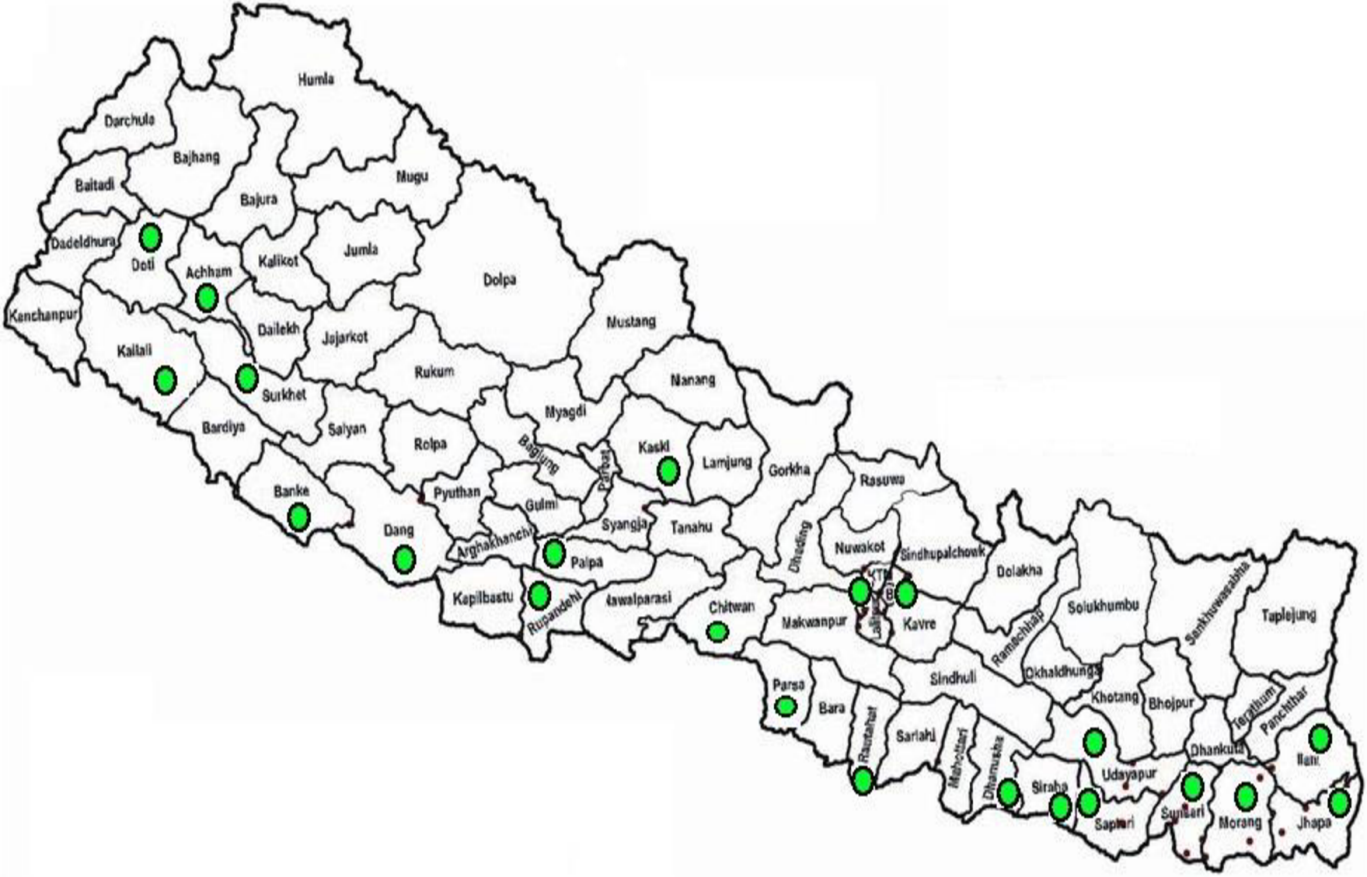
Districts equipped with Gene Xpert MTB/RIF centres in Nepal by 2015.

Nepal is a landlocked country located in South Asia with a total area of 147,181 km^2^ and a population of 26,494,405 [18]. The National Tuberculosis Centre (NTC) has five regional points under the Regional Health Directorate, and 75 district-level points under the District Health office (DHO) or District Public Health Office (DPHO) throughout the country. District hospital and primary health care centres (PHCs) are the basic health management unit for TB diagnosis and treatment in a district. Directly observed treatment short course (DOTS) is available at Health Posts and other health institutions (PHCs, hospitals, urban health clinics) within the district. Under the TB Reach project, 26 Gene Xpert machines have been installed since 2011 by the NTP and non-governmental organizations (NGOs) to provide free diagnostic services in government health facilities (DPHO laboratory, hospitals, PHCs), located throughout the country (Fig 1), but particularly in the urban setting. After completion of the TB Reach project in 2014/15, the NGOs donated the machines to the respective health facilities under NTP. This study was conducted between March-June 2017 and included all 26 diagnostic centres in Nepal with a Gene Xpert MTB/RIF machine installed before end of 2015.

There are 604 microscopic centres for TB diagnosis throughout the country established by the NTP. Two sputum specimens (morning and spot) are provided by presumptive TB patients for smear microscopy for acid-fast bacilli using Ziehl Neelsen technique in the majority of the centres. An additional single sputum specimen is collected from patients for the Xpert MTB/RIF assay in those centres with Gene Xpert MTB/RIF facility. Diagnosis in Xpert centres was done as described previously [19]. The priority patients for Xpert MTB/RIF test include the abovementioned WHO recommendations or additional presumptive TB patients referred to the centre by a physician. Extra-pulmonary (non-sputum) samples are also tested by Xpert MTB/RIF but the exact numbers are not known. The vast majority of samples tested were sputum.

### Study design

**Fig 2:**
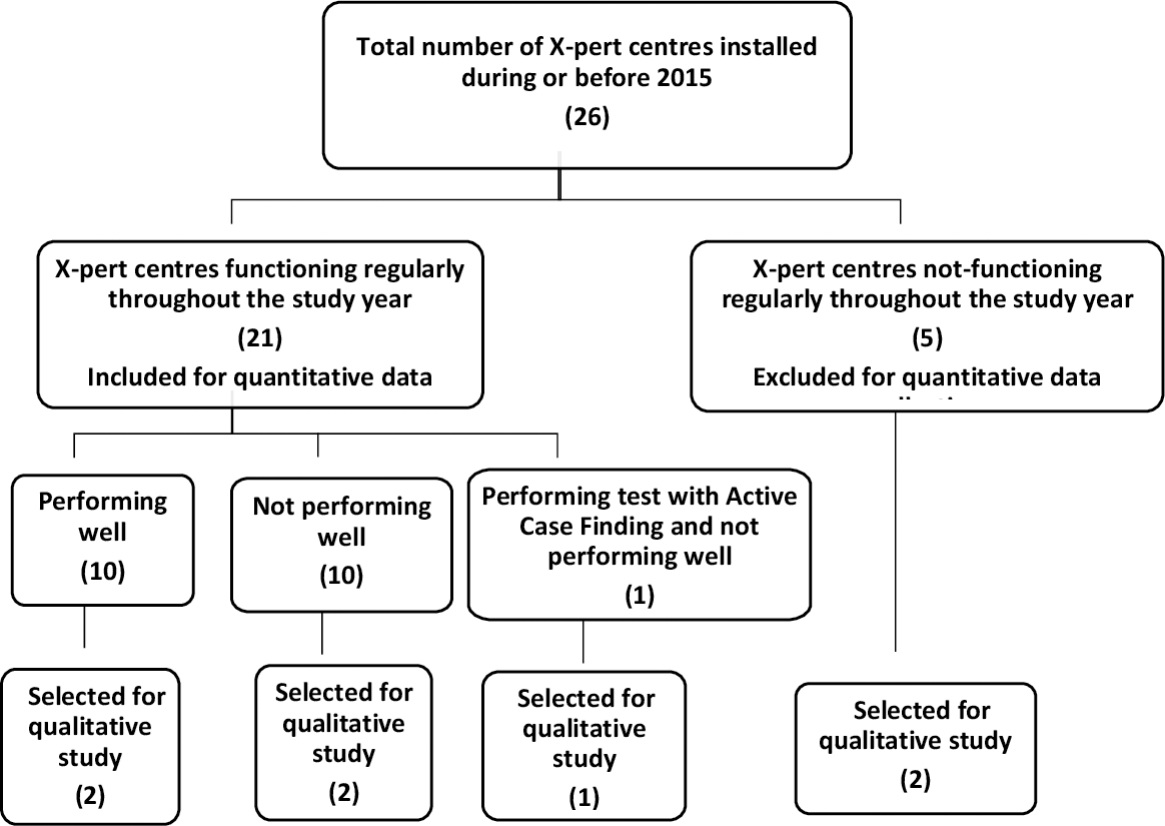
Flowchart for selection of centres for quantitative and qualitative data collection.

This was an implementation research using mixed methods sequential explanatory design. A quantitative analysis retrospectively evaluated the performance of the Xpert MTB/RIF assay at the diagnostic centres, and this was followed by a qualitative evaluation to explore the barriers to effective implementation of the Xpert MTB/RIF assay. Among all 26 Gene Xpert MTB/RIF centres installed in Nepal before 2015, 21 (81%) were regularly performing the assay throughout the one year period of evaluation: 17 July 2015 to 16 July 2016. For the quantitative study, data from all cases diagnosed by Xpert MTB/RIF assay over that one-year period were captured from these 21 Gene Xpert centres. There were no quantitative data available from the remaining 5 centres as they were not regularly performing Xpert assays over the study time period. Two of these five non-functional centres were purposively selected for the qualitative study. Fig 2 summarises selection of diagnostic centres.

### Data collection

Quantitative data were captured from the Gene Xpert MTB/RIF register and the general laboratory register which includes smear microscopy results from 21 functioning centres using a pre-tested data collection form to record results of Gene Xpert (positive, negative, test failure) and smear microscopy (positive, negative, not done) for comparison. We used the criteria test failure (error, invalid, no result) to distinguish between good performers and poor performers. As the maximum test failure rate was 13.4%, we set a cut-off value of 7% for test failure rate ≤7% as a good performer and >7% as a poor performer. This cut-off criteria is in agreement with multi-centre research reports conducted elsewhere which demonstrated an average test failure rate of around 7% [17,19,20]. After analysis of quantitative data, seven Gene Xpert centres were selected for the qualitative data collection, two that were performing well and five that were not performing well. The ‘not performing well centres’ (n = 5) included two of the five non-functioning Xpert centres, one each from plain and hilly region of the country.

Interviews were conducted with 22 presumptive TB patients who provided sputum for Xpert MTB/RIF assay, and these included 2 patients negative for a diagnosis of TB in comparison to 20 patients diagnosed with TB. This is because the Xpert results are usually collected by the patient’s family members or legally authorized persons during the result distribution day, but not by the patient themselves, and so the positive cases were interviewed once treatment was started in the DOTS clinic which was not possible for negative cases. Interviews were undertaken in the local language, using a semi-structured format with open-ended questions. Interview guides are depicted in supporting information section (S1 and S2 Files). Questions explored the following themes: knowledge and beliefs about TB transmission and disease, perceived barriers for diagnosis and treatment; and experience in using the diagnostic centre.

Four FGDs were conducted in the local language with the district TB officer and/or lab personnel in four centres. In two centres, the FGDs were replaced by IDIs due to small numbers of participants. A thematic interview/FGD guide with broad and open-ended questions covered the following main themes: (1) experience with the use of Xpert MTB/RIF diagnosis and compared to smear; (2) communication of results to patients and TB officer responsible for care; (3) challenges in specimen collection and results; and (4) challenges for supply and maintenance of laboratory equipment. At the national level, IDIs were conducted with four informants, i.e. NTP focal person for Xpert MTB/RIF, monitoring and evaluation section chief, WHO focal person for NTC and TB coordinator for International Organization of Migration (IOM). IDIs and FGDs were recorded except for nine interviews where recordings were not permitted.

### Data analysis

Quantitative data were double entered, validated and analysed using Microsoft Excel™. Descriptive statistics were used for quantitative variables. Error rate and positivity rate for each particular centre were determined by calculating percentage of total tests done in that centre during the study period. The proportion of indeterminate test results was calculated as per 100 tests. The number of tests per module per day was derived from the collected data assuming 24 working days in a month. Kappa score was calculated to determine the strength of agreement between the results from Xpert and smear microscopy.

For qualitative data, transcripts from recordings were available within 24 hours. Personal identities were removed in the transcripts to ensure confidentiality. These transcriptions and completed notes from unrecorded interviews were then translated into English by the first author and note takers, and cross-checked. The transcription, translation and cross-check were carried out immediately after data collection to ensure data credibility and enhance reliability of the interpretations. The English version transcriptions were imported into Open Code 4.03 qualitative software. Codes were created and were repeatedly used to the data sets through continuous comparison within and across the transcripts in developing inductive codes. Synthesis of codes was done, and themes were generated. Inductive codes were used for writing narratives.

### Ethics approval

Ethical approval for this study was obtained from the Nepal Health Research Council (NHRC), Nepal (Reg no. 39/2017). Permission from NTP authorities and Gene Xpert centres were also obtained. Written informed consent was obtained from the study participants.

## Results

### Profiles of Gene Xpert MTB/RIF centres

Of 26 Gene Xpert centres installed up to 2015, 21 performed tests regularly throughout the year. These functioning centres were operated by different institutions as follows: 10 by IOM, 7 by NTC, 3 by the Health Research and Social Development Forum (HERD) and 1 by German Nepal TB project (GENETUP) that supply cartridge, calibration and maintenance. HERD was the only operator providing mobile centres in a van. The 5 non-functioning centres were all managed by the NTC.

### Quantitative results

A total of 23,075 tests were performed in 21 Gene Xpert centres in one year, ranging from 201 to 3,976 tests (Table 1). Overall, Xpert MTB/RIF assay was positive in 18% (range 6.5–49%) of tests and the proportion that were Rifampicin resistant (RR) positive of those that were Xpert positive was 8.2% (range 3.7–21%).

**Table 1:** Annual performance scores of Gene X-pert MTB/RIF centres in Nepal, 2015–2016.

No.	Code of the Centre	Ope- ra tor	Total no. of test done	No. of positives by Gene X-pert MTB/RIF				Total test failure			No. of test failure (%)	Proportion of indeterminate result (%)	No. of tests per module per day
				RS	RR(%)#	RI	Total	SP	SN	SND			
**1**	X1	NTC	1020	276	19 (6.4)	4	299 (29.3)	6	41	2	49 (4.8)	1.3	0.9
2	X2	GENETUP	470	181	24 (11.7)	1	206 (43.8)	6	24	0	30 (6.4)	0.5	0.4
**3**	X3	IOM	1,648	277	26 (8.5)	2	305 (18.5)	3	76	0	79 (4.8)	0.7	1.4
4	X4^a^	IOM	2,305	341	16 (4.4)	6	363 (15.8)	2	66	20	88 (3.8)	1.7	4.0
**5**	X5	IOM	1,856	281	16 (5.4)	1	298 (16.1)	0	76	0	76 (4.1)	0.3	1.6
6	X6^a^	IOM	801	47	5 (9.6)	0	52 (6.5)	0	33	0	33 (4.1)	0.0	1.4
**7**	X7^a^	IOM	730	58	6 (9.2)	1	65 (8.9)	0	19	0	19 (2.6)	1.5	1.3
8	X8	IOM	1,918	503	35 (6.4)	11	549 (28.6)	3	104	0	107 (5.6)	2.0	1.7
**9**	X9	IOM	1,123	157	11 (6.5)	1	169 (15.1)	0	55	0	55 (4.9)	0.6	1.0
10	X10	IOM	1,196	151	6 (3.7)	4	161 (13.5)	0	62	0	62 (5.2)	2.5	1.0
**11**	X11	NTC	449	97	17 (14.4)	4	118 (26.3)	0	38	0	38 (8.5)	3.4	0.8
12	X12	NTC	283	41	6 (12.0)	3	50 (17.7)	3	30	0	33 (11.7)	6.0	0.5
**13**	X13	HERD	158	49	6 (10.7)	1	56 (35.4)	1	20	0	21 (13.3)	1.8	0.3
14	X14	NTC	745	186	21 (10.0)	2	209 (28.1)	6	36	21	63 (8.5)	1.0	1.3
**15**	X15	NTC	414	155	43 (21.2)	5	203 (49.0)	9	41	1	51 (12.3)	2.5	0.7
16	X16	NTC	743	201	48 (19.0)	4	253 (34.1)	7	70	0	77 (10.4)	1.6	0,6
**17**	X17	NTC	201	33	7 (17.1)	1	41 (20.4)	0	27	0	27 (13.4)	2.4	0.3
18	X18	IOM	1,188	151	6 (3.8)	2	159 (13.4)	1	98	0	99 (8.3)	1.3	2.1
**19**	X19	IOM	1,547	388	19 (4.6)	3	410 (26.5)	0	166	8	174 (11.3)	0.7	1.3
20	X20^b^	HERD	304	53	6 (10.4)	1	60 (19.7)		28		28 (9.2)	1.7	0.5
**21**	X21	HERD	3,976	299	12 (3.8)	8	319 (8.0)	0	531	0	531 (13.4)	2.5	1.2
	**Total**		23,075	3,925	355 (8.2)	65	4,345 (18.3)	47	1,641	52	1,740 (7.5)	1.5	1.1

^a^—Data upto April 30 2016

^b^—17 Nov 2014 to 16 nov 2015 and total error percentage

# proportion rifampicin resistant positive of total X-pert positive results; RS: Rifampicin Sensitive, RR: Rifampicin Resistant; RI: Rifampicin Indeterminate; SP: Smear Positive; SN: Smear Negative; SND: Smear Not Done

Ten of the 21 centres had a test failure rate of ≤ 7% and the highest test failure rate was 13.4%, observed in two centres. The proportion of indeterminate results ranged from 0–6%. Nineteen centres performed less than two tests per module per day. Based on the test failure rate, positivity rate and proportion of indeterminate results, we categorised these 21 functioning centres as good performers (10 centres listed number 1–10) and poor performers (11 centres listed number 11–21), in addition to the 5 non-functioning centres.

Of the 23,075 tests performed, 22,288 samples (96.6%) were tested by both diagnostic tools, i.e. Xpert and smear microscopy (Table 2). Among these samples, 15% were Xpert positive and smear microscopy negative, while only 113 (0.5%) samples were smear microscopy positive and Xpert negative. There was concordance between the two diagnostic tools for both positive and negative results in 77% of cases. The overall agreement between the two tests was fair, with Kappa score of 0.3 (95% CI 0.28,0.32).

**Table 2:** Gene X-pert MTB/RIF and Microscopy results by testing centres in Nepal, 2015–2016.

No	Code of the Centre	Type of facility		Total sample tested by both method	Positive by both smear and X-pert (%)	Negative by both smear and X-pert (%)	X-pert positive and smear negative (%)	X-pert negative and smear-positive (%)	N test failure (%)	Kappa score (95%CI)
**1**	X1	NTC		980	53 (5.4)	639 (65.2)	235 (24.0)	6 (0.6)	47 (4.8)	0.2 (0.12–0.28)
**2**	X2	TB reference Lab		468	150 (32.1)	219 (46.8)	55 (11.8)	14 (3.0)	30 (6.4)	0.7 (0.63–0.77)
3	X3	NGO		1642	55 (3.3)	1252 (76.2)	249 (15.2)	7 (0.4)	79 (4.8)	0.2 (0.12–0.28)
**4**	X4^a^	Teaching Hospital		1943	135 (6.9)	1568 (80.7)	166 (8.5)	6 (0.3)	68 (3.5)	0.6 (0.54–0.66)
5	X5	Hospital		1856	0 (0.0)	1465 (78.9)	298 (16.1)	17 (0.9)	76 (4.1)	0.0 (-0.10–0.10)
**6**	X6^a^	DHO		800	5 (0.6)	715 (89.4)	47 (5.9)	0 (0.0)	33 (4.1)	0.2 (-0.03–0.43)
7	X7^a^	DHO		730	6 (0.8)	646 (88.5)	59 (8.1)	0 (0.0)	19 (2.6)	0.2 (-0.01–0.41)
**8**	X8	NTC		1849	86 (4.7)	1200 (64.9)	427 (23.1)	29 (1.6)	107 (5.8)	0.2 (0.14–0.26)
9	X9	PHC		1122	4 (0.4)	897 (79.9)	165 (14.7)	1 (0.1)	55 (4.9)	0.1 (-0.04–0.23)
**10**	X10	Hospital		1189	14 (1.2)	967 (81.3)	146 (12.3)	0 (0.0)	62 (5.2)	0.1 (-0.03–0.23)
11	X11	DPHO		449	4 (0.9)	293 (65.3)	114 (25.4)	0 (0.0)	38 (8.5)	0.1 (-0.05–0.25)
**12**	X12	Hospital		269	14 (5.2)	183 (68.0)	35 (13.0)	4 (1.5)	33 (12.3)	0.4 (0.21–0.59)
13	X13	DPHO		158	19 (12.0)	80 (50.6)	37 (23.4)	1 (0.6)	21 (13.3)	0.4 (0.23–0.57)
**14**	X14	Regional TB Centre		515	56 (10.9)	312 (60.6)	89 (17.3)	16 (3.1)	42 (8.2)	0.4 (0.30–0.50)
15	X15	Hospital		412	139 (33.7)	149 (36.2)	63 (15.3)	11 (2.7)	50 (12.1)	0.6 (0.52–0.68)
**16**	X16	Hospital		743	49 (6.6)	413 (55.6)	204 (27.5)	0 (0.0)	77 (10.4)	0.2 (0.11–0.29)
17	X17	Hospital		195	0 (0.0)	130 (66.7)	38 (19.5)	0 (0.0)	27 (13.8)	0.0 (-0.28–0.28)
**18**	X18	PHC		1185	21 (1.8)	926 (78.1)	138 (11.6)	1 (0.0)	99 (8.4)	0.2 (0.08–0.23)
19	X19	Hospital		1509	15 (1.0)	938 (62.2)	390 (25.8)	0 (0.0)	166 (11.0)	0.0 (-0.08–0.08)
20 21	X20^b^	DHO		298	19 (6.4)	210 (70.5)	41 (13.8)	0 (0.0)	28 (9.4)	0.5 (0.34–0.66)
	X21	NGO		3976	1 (0.0)	3126 (78.6)	318 (8.00	0 (0.0)	531 (13.4)	0.0 (-0.10–0.10)
	**Total**			**22288**	**845 (3.8)**	**16328(73.3)**	**3314 (14.9)**	**113 (0.5)**	**1688 (7.6)**	**0.3 (0.28–0.32)**

^a^—Data to April 30, 2016

^b^—17 Nov 2014 to 16 nov 2015

Regarding overall performance of the operators, test failure rates ranged between 5.5% and 12% (Table 3). Similarly, average positivity rate was found to be highest for NTC (29.3%) and lowest for IOM (16.3%). For GENETUP, error and positivity rate were 6.4% and 43.8% respectively (Table 3). The number of cartridges used for one positive case to diagnose was maximum for HERD (10.2) and minimum for GENETUP (2.3). Nearly 8% of the people who were positive by smear microscopy were found to be indeterminate by Xpert. For indeterminate results, Xpert centres perform second Xpert MTB/RIF test from new sputum sample.

**Table 3:** Average annual performance score of centres according to their operators in Nepal, 2015–2016.

Operator	Number of centres operated	Average test failure rate	Total number of cartridges used	Average positivity rate	Cartridge spent to find one positive case
**GENETUP**	1	6.4	470	43.8	2.3
**HERD**	3	12.0	4438	21.6	10.2
**IOM**	10	5.5	14312	16.3	5.7
**NTC**	7	9.9	3855	29.3	3.3

### Qualitative findings

During thematic analysis of qualitative data, we found barriers and facilitators for implementation and scale up as main theme comprising of two sub themes i.e. 1. Barriers to the use of Xpert MTB/RIF and 2. Facilitators for implementation and scale up of Xpert MTB/RIF.

All laboratory personnel found Xpert MTB/RIF as a novel diagnostic tool, superior to smear microscopy. ‘*Gene Xpert can detect TB in low bacilli load so it is superior to microscopy*.’ (Xpert staff). A rapid result in a few hours and the ability to detect drug resistant TB early by giving the status of Rifampicin resistance in positive cases were also mentioned by the Gene Xpert lab staff. For the vulnerable group for testing, the health staff working in ART centres stated that they refered people living with HIV (PLHIV) and sputum smear-negative cases for Xpert MTB/RIF testing. All stakeholders emphasized that the diagnosis of all presumptive cases of TB by Xpert should not replace microscopy and culture. ‘*AFB or culture or Gene Xpert cannot replace each other but supplementary to other tests*. *Gene Xpert is a novel diagnostic tool for TB*, *but it has drawbacks like it can detect dead bacteria so it cannot be used for follow up cases*. *So microscopy and Gene Xpert is necessary*.’ (Gene Xpert staff). Gene Xpert centres could not reach sufficient vulnerable groups. MDR suspects and some PLHIV were diagnosed with TB by Xpert, but very few children were diagnosed due to low referral of children for Xpert assay. During active case finding targeting specific vulnerable groups, they achieved more tests for vulnerable groups. ‘*We have very less coverage for PLHIV and children but more coverage in case of MDR suspect*.’ (X-pert staff)

Patients also prefer Xpert test thinking it will give an accurate result because it is computer-based. ‘*They will go for test (i*.*e*. *Gene X-pert)*. *Patients demand to test by machine/ computer*. *They have trust towards Gene X-pert*. *Even though we only test by X-pert if referred by physician*.’ (X-pert staff) We found that all of the MDR TB cases who we interviewed were first diagnosed by Xpert MTB/RIF assay followed by culture and DST. Barriers during implementation of Xpert MTB RIF assay for the diagnosis of TB are summarised in Table 4.

**Table 4:** Barriers to implementation.

Barriers for implementation	Participants’ views	
	Agreement	Disagreement
**Poor laboratory infrastructure**	‘*When we implemented to set up*, *most laboratories in Nepal were not suitable for Gene Xpert*. *NTC supported laboratories do not have well-established set-up*. *Dusty environment*, *no proper furniture*, *crowded room*.’ (IDI participant). *‘Dust and high temperature during summer increased the number of test failure result*.*’* (IDI participant)	
**Need of incentive for staff**	*‘More incentives should be given to laboratory personnel because we have more workload*.*’ (FGD and IDI participants)*	‘*Issue of incentives were a big problem*. *Though it looked attractive in the beginning*, *it demoralized the program*. *It was a big hurdle for the expansion*.’ (FGD participant)
**Delays in cartridge supply, calibration and replacement of module**	Many peripheral level staff working in Gene Xpert consistently reported that delays in supply of cartridge, calibration and replacement of damaged modules were a challenge for implementation.	‘*District should ask for the materials at least before 15 days or at least half of the stock is finished*. *Intentionally some people request for the stocks in last hours*.’ (FGD participant from NTC).
**Long travelling distance for patients**	‘*Hospital is so far so I have travelled in the vehicle and it is difficult for me*.’ (IDIP 15).	

Interruption of power supply due to load shedding was identified as a barrier in majority of centres for running Xpert machines. To overcome supply chain and management issue NTC has assigned focal persons for Gene Xpert centres in each region. ‘*We have established Biomedical unit for solving Gene Xpert problems*. *From this year office of supplier will be established in Nepal which will make it easy to solve the problems related to Gene X-pert machine*.’ (NTC supervisor).

### Facilitators for implementation and scale up of Xpert MTB/RIF

Some centres suggested NTC to decentralize the manpower for calibration. During FGD with NTC laboratory personnel, they suggested giving Training of Trainers for maintenance and calibration of the machine so that peripheral staff have the capability for calibration and handling minor problem in Gene Xpert machines. They also suggested for maximum use of Xpert for the vulnerable population. ‘*Should be patient-centred- samples should be received on the spot so that patient will get the result in time and they can save their money as they come here from many other districts*.’ (Xpert staff). Timely replacement of the non-functional modules and increase in the modules to increase capacity are common suggestions from FGD participants. Some of the participants also suggested conducting community awareness as well as awareness to health staff so that eligible patients should be referred for Xpert test and follow-up patients should not be referred for Xpert. Other IDI participant said that Nepal is not utilizing Gene Xpert facility because many modules are not working.

Practical feasibility planning should be done for scale-up of Gene Xpert technology. All participants supported the expansion of Gene Xpert machines to all districts of Nepal. NTP also plans to install a Gene Xpert machine in all 75 districts by 2020. Some participants suggested conducting research before planning for scaling up. Commitment and ownership should be taken from the government. ‘*We should be more focused on stability and maintenance before expanding Xpert machine*.’ (FGD participant).

## Discussion

In our study, Xpert MTB/RIF assay detected a higher number of bacteriologically confirmed TB cases than sputum microscopy which supports the findings presented elsewhere [21]. Nepal NTP reported that Xpert MTB/RIF diagnosed 28% of the total bacteriologically confirmed TB cases in 2015/16 [5]. This indicates the importance of this technology for increased case detection. However, this mixed method implementation research study found that more than half of the Gene Xpert centres were not performing well. The main reasons for weak implementation for Gene X-pert was found to be ineffective supply chain management with delays in supply of cartridges as well as replacement of damaged modules and maintenance from NTC. There was inadequate systematic stock verification to inform timely procurement of cartridges by the Gene Xpert centres and a lack of communication between NTC and Gene Xpert centres. Poor infrastructure in the laboratory where Gene Xpert machines are installed also plays a significant role in weak implementation.

Test results from the majority of Xpert assays had a fair agreement with sputum smear microscopy results. The finding that 15% of patients were Xpert positive for MTB and sputum smear-negative is similar to findings from India of 18% [22]. A previous study conducted in Nepal of intensified case finding of TB cases in vulnerable populations reported that 7% were Xpert positive and smear-negative [10]. A study conducted in 18 countries among TB/HIV co-infected patients reported 23% cases positive by Xpert and negative by smear microscopy [23].

In our study, 11 of 21 centres had test failure rates of more than 7% and 2 out of 21 centres had high rates of indeterminate results. Reasons for this were unclear but may indicate poor sputum collection technique or environmental impact on cartridges such as the high temperature of the laboratory which was also found in a study from Mongolia [24]. The overall average test failure rates of 7.5% was less than that reported (10.7%) in one research conducted in nine countries, including Nepal [21]. High test failure rate and indeterminate results observed in the centres may also be due to poor laboratory infrastructure, frequent power cuts, lack of air conditioners and/or dusty environment. Uninterrupted power supply and provision for controlling temperature of the laboratory room are necessary for running Gene Xpert machines. Research report finds constant power supply as a challenge for implementation of Xpert MTB/RIF for the diagnosis of TB in developing countries [25]. Environmental factors, such as dust, are also found to be a cause of module failure [19]. Proper instruction for quality sputum collection from presumptive cases by lab staff may minimize error rates from Gene Xpert tests. Similarly, physicians and other health workers should be informed about the type of suspected patients that should be referred for Gene Xpert test.

We observed that one staff member in each laboratory was appointed as the focal person for Xpert MTB/RIF testing. However, during FGD we learnt that the majority of staff from Gene Xpert centres had demanded incentives for their increased workload due to Xpert MTB/RIF assay. Therefore, a timely review by NTP to evaluate actual additional workload for staff due to Xpert testing in the laboratories may be helpful. For improving the quality of testing from Xpert MTB/RIF, comprehensive training of a minimum of five days to the staff of Gene Xpert centres is necessary with a focus on materials and practicals on the optimal use of Gene Xpert technology [26]. Similarly, regular monitoring and evaluation of Gene X-pert sites are also equally important [26]. As we observed in Nepal, calibration of machines and replacement of damaged modules are major challenges for low and middle-income countries [19,21]. These challenges can be overcome by co-operation between staff working for Gene Xpert, operator and supplier to minimize delay in demand from Gene Xpert centre and minimize delay for supply, as well as maintenance from operator and supplier side.

The strength of this study was the performance analysis of a large number of Gene Xpert centres and selection of centres for qualitative study by their performance. For quantitative research, we used performance indicators of Xpert MTB/RIF. Qualitative research further explored the reason for performance of Gene X-pert and steps to be taken for optimal performance. Our study has broad coverage within the country, so the study findings can be generalized. We used reporting guidelines for implementation and operational research, Standards for Reporting for Implementation Studies (StaRI) [27,28].

There are important limitations in this study. We used secondary data for quantitative research and there may have been errors in data recording in Gene Xpert registers. Due to lack of culture facility in majority of the Gene Xpert centres, we could not compare the results from culture and Xpert which would have added value for comparison. For test failure and indeterminate results, we did not trace the outcomes for those results, which may be necessary for analysing the exact number of people who did not get the interpretable result (positive or negative) from Gene Xpert technology. This would also be important for assessing the correct number of people benefited from Gene X-pert facility.

## Conclusion

Xpert MTB/RIF testing has increased case-finding of TB and MDR TB in Nepal. However, the performance of the existing diagnostic centres needs to be improved, and this study has identified challenges to optimize future scale-up of Gene Xpert centres in Nepal.

## Supporting information

S1 File Interview Guide_ English. (DOCX)

S2 File Interview Guide_ Nepali. (DOCX)

S3 File Dataset. (XLSX)
