# Supplementary material for: The implementation of Xpert MTB/RIF assay for diagnosis of tuberculosis in Nepal: A mixed-methods analysis
Source: PLoS One. 2018 Aug 10;13(8):e0201731. doi: 10.1371/journal.pone.0201731 (PMC6086427; doi:10.1371/journal.pone.0201731)
Supplement: S1 File — (DOCX) [file pone.0201731.s001.docx]

**The implementation of Xpert MTB/RIF assay for diagnosis of tuberculosis in Nepal: a mixed-methods analysis**

# FGD guideline stakeholders

Date:

Code no.:

Introduction for the interviewee

Name:

Sex:

Designation:

District:

Experience in current organization:

Experience in related field:

1. Can you provide brief information on the current situation of TB and HIV in this area? (Probe: number of cases, high risk groups, case detection rate, treatment success rate )
2. What kind of programs are being carried out regarding TB diagnosis in this district?
3. How are you planning for the early diagnosis of TB in this district? ( diagnosis centers, active case findings)
4. Do you want to share your experience while using gene Xpert MTB/RIF as diagnostic tool for TB?
5. Which diagnostic tool (gene Xpert MTB/RIF or sputum smear microscopy) do you find more easy for diagnosing TB?
6. What are the major problems being seen for the implementation of gene Xpert MTB/RIF (Power supply, cartridge supply, maintenance facility from NTP) ?
7. Do you think high risk groups (PLHIV, children, relapse cases) are willing to involve for the diagnosis of TB by gene Xpert MTB/RIF in your laboratory?
8. In your opinion how can we increase case detection of TB by using gene X-pert MTB/RIF technology?
9. At last do you have any suggestions for solving the weak implementation of gene X-pert MTB/RIF and increasing case detection rate by using the novel diagnostic tool? (Not more than 5 points)

THANK YOU

**The implementation of Xpert MTB/RIF assay for diagnosis of tuberculosis in Nepal: a mixed-methods analysis**

# IDI with TB patients diagnosed by gene X-pert MTB/RIF

Date:

Code no.:

Introduction for the interviewee

Name:

Age (Sex):

Address:

1. What do you think is the cause of TB?
2. From where and by whom are you referred for the diagnosis of TB?
3. Are you satisfied with the service provided by health workers for TB diagnosis in your district?
4. Have you heard about TB diagnosis by gene Xpert MTB/RIF?
5. Which diagnostic tool gene Xpert MTB/RIF or sputum smear do you find easier for diagnosing TB?
6. Do you find the diagnosis of TB by gene Xpert MTB/RIF more reliable, feasible and fast than sputum smear test? If yes, in what ways has this diagnosis method helped you?
7. Did you benefit by the early test results by gene Xpert MTB/RIF? If yes, what are the benefits you have experienced?
8. Are there any disadvantages or difficulties (assess to center, time to get result) you have faced during diagnosis of TB by gene Xpert MTB/RIF?
9. Do you have any suggestions to say regarding gene Xpert MTB/RIF facility?

THANK YOU

**The implementation of Xpert MTB/RIF assay for diagnosis of tuberculosis in Nepal: a mixed-methods analysis**

# IDI with stakeholder- IOM/GENETUP/HERD

Date:

Code no.:

Introduction for the interviewee

Name:

Sex:

Designation:

Experience in current organization:

Experience in related field:

1. Can you provide brief information on the current programs by IOM/GENETUP/HERD in Nepal?
2. What kind of programs are being carried out regarding TB and HIV diagnosis by IOM/GENETUP/HERD in Nepal? (Probe: preventive, curative)
3. How IOM/GENETUP/HERD is helping Nepal NTP for the early diagnosis of TB and what are the plans of IOM/GENETUP/HERD for improving and early diagnosis of TB in Nepal? (diagnosis centers, active case findings)
4. While you implemented gene Xpert MTB/RIF assay in Nepal, which group did you find vulnerable for TB?
5. What are the major problems being seen for the implementation of gene Xpert MTB/RIF in Nepal?
6. Do you think high risk groups are involved in the program for the diagnosis of TB by gene Xpert MTB/RIF in your district?
7. What are your suggestions for increase case detection of TB by using gene Xpert MTB/RIF technology?
8. In your opinion, what plan of actions should NTP adopt to scale up gene Xpert MTB/RIF for routine diagnosis of TB?

THANK YOU

**The implementation of Xpert MTB/RIF assay for diagnosis of tuberculosis in Nepal: a mixed-methods analysis**

# IDI with laboratory staff

Date:

Code no.:

Introduction for the interviewee

Name:

Sex:

Designation:

Experience in current organization:

Experience in related field:

1. Can you provide brief information on the current situation of TB and HIV in this

area?

1. What kind of programs is being carried out regarding diagnosis of TB in your centre?
2. How are you planning for the early diagnosis of TB in this center?
3. Do you want to share your experience while using gene Xpert MTB/RIF as diagnostic tool for TB?
4. Which diagnostic tool (gene Xpert MTB/RIF or sputum smear microscopy) do you find more easy for diagnosing TB?
5. What are the major problems being seen for the implementation of gene Xpert MTB/RIF (Power supply, cartridge supply, maintenance facility from NTP)?
6. Do you think high risk groups (PLHIV, children, relapse cases) are willing to involve for the diagnosis of TB by gene Xpert MTB/RIF in your laboratory?
7. In your opinion how can we increase case detection of TB by using gene Xpert MTB/RIF technology?
8. At last do you have any suggestions for solving the weak implementation of gene Xpert MTB/RIF and increasing case detection rate by using the novel diagnostic tool? (Not more than 5 points)

THANK YOU

**The implementation of Xpert MTB/RIF assay for diagnosis of tuberculosis in Nepal: a mixed-methods analysis**

# IDI with NTP focal person

Date:

Code no.:

Introduction for the interviewee

Name:

Sex:

Designation:

Experience in current organization:

Experience in related field:

1. Can you provide brief information on the current national scenario of TB in Nepal? (Probe: number of cases, high risk groups, case detection rate, treatment success rate )
2. What kind of programs are being carried out from this centre for TB and HIV control? (Probe: preventive, curative)
3. How are you planning for the early diagnosis of TB? ( diagnosis centers, active case findings)
4. Do you want to share your experience while implementing gene Xpert MTB/RIF programs for the diagnosis of TB?
5. Which group are vulnerable for TB in Nepal?
6. What are the major health problems being seen for the implementation of gene Xpert MTB/RIF in Nepal?
7. Do you think high risk groups are involved in the program for the diagnosis of TB by gene Xpert MTB/RIF?
8. What is your plan for increasing case detection of TB by using gene Xpert MTB/RIF technology?
9. What is NTP’s plan to scale up gene Xpert MTB/RIF for routine diagnosis of TB?

THANK YOU

**The implementation of Xpert MTB/RIF assay for diagnosis of tuberculosis in Nepal: a mixed-methods analysis**

# IDI with WHO staff

Date:

Code no.:

Introduction for the interviewee

Name:

Sex:

Designation:

Experience in current organization:

Experience in related field:

1. Can you please give brief information about current situation of TB and HIV?
2. What kind of programs is being carried out regarding TB and HIV by WHO?
3. How WHO is helping in making planning in early diagnosis of TB in Nepal?
4. Do you want to share experiences while implementing gene Xpert MTB/RIF for the diagnosis of TB?
5. According to WHO which groups are Vulnerable for TB in Nepal?
6. What are the major problems for effective implementation of Gene Xpert in Nepal?
7. Are vulnerable groups are diagnosed for TB by Xpert technology?
8. Do you have any suggestion and Recommendation for effective implementation of Xpert MTB/RIF technology for diagnosis of TB in Nepal?

THANK YOU

**The implementation of Xpert MTB/RIF assay for diagnosis of tuberculosis in Nepal: a mixed-methods analysis**

# Informed Consent

Namaskar! I am Basant Joshi, a student of Master of Public Health in Gadjah Mada University, Indonesia. As a partial fulfillment of the requirement of Master of Public Health I am going to conduct research on **“The implementation of Xpert MTB/RIF assay for diagnosis of tuberculosis in Nepal: a mixed-methods analysis”.** I need to take information from you as an important respondent of my study. Your name has nothing to do with the research findings and your name will never be published anywhere. If you are interested to participate I will proceed, otherwise I will leave and won’t be disappointed if you are not interested. Your contribution can make a difference to make a clear picture of the problem. The information given by you will only be used for the fulfilment of the research objective. I will be grateful if you will help me in this regard.

May I proceed?

If yes, start………………………………………………………………...

…………………… Signature of the respondent

…………………… Signature of the researcher

Date : …………………………………
