# Supplementary material for: The implementation of Xpert MTB/RIF assay for diagnosis of tuberculosis in Nepal: A mixed-methods analysis
Source: PLoS One. 2018 Aug 10;13(8):e0201731. doi: 10.1371/journal.pone.0201731 (PMC6086427; doi:10.1371/journal.pone.0201731)
Supplement: S2 File — (DOCX) [file pone.0201731.s002.docx]

**;/f]sf/jfnfx?;Fusf] FGD**

ldlt M

sf]8 g+M

cGt/jf{tf lng] JolQmsf] kl/ro

gfd M

lnË M

kb M

lhNnf M

xfnsf] ;+:yfdf cg'ej M

;DalGwt lgsfodf cg'ej M

!= s] tkfO{n] o; If]qsf] l6=aL= / Pr=cfO=eL= sf] jt{dfg cj:yfaf/]df hfgsf/L lbg ;Sg'x'G5<

-s];sf] ;+Vof, hf]lvd ;d"x, s]; kQf nufpg] b/, ;kmn pkrf/ b/_

@= o; lhNnfdf l6=aL= lgbfgsf nflu s] s:tf lsl;dsf sfo{s|dx? ;+rfng eO/x]sf 5g\ <

#= tkfO{n] lhNnfdf ;dofdfg} l6=aL= lgbfg ug{sf] nflu s] s:tf of]hgfx? agfpg'ePsf] 5 <

-lgbfg s]Gb|x?, active case findings)

$= l6=aL= lgbfg ug{sf] nflu lhg PS;k6{ k|of]u ubf{sf] tkfO{sf] cg'ej s:tf] /x\of] <

%= l6=aL= lgbfg ug{sf] nflu s'g kl/If0f ljlw a9L ;lhnf] dfGg'x'G5 < -lhg PS;k6{ jf dfOs|f]:sf]kåf/f vsf/ kl/If0f_

^= lhg PS;k6{åf/f kl/If0f ug{] sfo{ sfof{Gjog ug{sf] nflu b]lVfPsf d'Vo ;d:ofx? s] s] x'g\<

-ljB't cfk"lt{, cartridge sf] cfk"lt{ /fli6«o Ifo/f]u s]Gb|åf/f dd{tsf] ;'ljwf_

&= hf]lvd ;d"xx? h:t} Pr=cfO=eL= ;ª|s|ldt JolQm, afnaflnsf cflb tkfO{sf] lhg PS;k6{ ljlwåf/f tkfO{sf] k|of]uzfnfdf kl/If0f ug{ sltsf] OR5's 5g\ <

*= tkfO{sf] ljrf/df lhg PS;k6{ ljlwsf] k|of]u u/]/ s;l/ Ifo/f]u kQf nufpg] ;+Vof a9fpg ;lsG5 <

(= cTodf lhg PS;k6{sf] lkmtnf] sfof{Gjogsf] ;d:of ;'Nemfpg / lgbfgsf] glag pks/0f sf] k|of]u u/]/ Ifo/f]uLsf]] ;+Vof a9fpg s] ;'emfj lbg rfxg'x'G5 <

**wGojfb**

**a/fdLx?;Fusf] cGt/jftf{**

ldlt M

sf]8 g+M

cGt/jf{tf lng] JolQmsf] kl/ro

gfd M

lnË M

kb M

lhNnf M

xfnsf] ;+:yfdf cg'ej M

;DalGwt lgsfodf cg'ej M

!= tkfO{sf] ljrf/df Ifo/f]u nfUg'sf] d'Vo sf/0f s] xf]nf <

@= tkfO{nfO{ Ifo/f]u hfFrsf] nflu s;n] k|]if0f u/]sf] xf] <

#= tkfO{sf] lhNnfdf :jf:YosdL{x?n] Ifo/f]u kl/If0fsf nflu k|bfg ug{] ;]jfaf6 tkfO{ ;Gt'i6 x'g'x'G5 <

$= s] tkfO{n] lhg PS;k6{ ljlwåf/f Ifo/f]u lgbfgsf] af/]df ;'Gg'ePsf] 5 <

%= Ifo/f]u lgbfgsf nflu lhg PS;k6{ ljlw / dfOs|f]:sf]kdf vsf/ kl/If0f ljlw dWo] tkfO{nfO{ s'g ljlw ;lhnf] nfU5 <

^= s] tkfO{nfO{ lhg PS;k6{ ljlwåf/f Ifo/f]u lgbfg dfOs|f]:sf]kdf vsf/ kl/If0f ljlweGbf a9L ljZj;gLo, ;:tf] / l56f] nfUb5 < olb nfU5 eg] ;f] ljlwn] tkfO{nfO{ s'g tl/sfn] ;xof]u u/]sf] 5 <

&= s] tkfO{nfO{ lhg PS;k6{ ljlwåf/f l56f] x'g] kl/If0fn] s]lx kmfObf kfpg' ePsf]5 < olb 5 eg] s:tf] lsl;dsf] kmfObfsf] dx;'; ug{'ePsf] 5 <

*= lhg PS;k6{ ljlwåf/f Ifo/f]u kl/If0f ug{] s|ddf s] tkfO{n] s'g} lsl;dsf] c;lhnf] dx;'; ug{'ePsf] 5 <

(= cGtodf lhg PS;k6{ ljlwåf/f Ifo/f]u kl/If0fsf] ;'ljwfsf] ;DaGwdf tkfO{nfO{ s]lx ;'emfj lbg dg nfu]sf] 5 <

**wGojfb**

**;/f]sf/jfnfx?;Fu cGt/jftf{ - IOM/ GENETUP/ HERD**

ldlt M

sf]8 g+M

cGt/jf{tf lng] JolQmsf] kl/ro

gfd M

lnË M

kb M

lhNnf M

xfnsf] ;+:yfdf cg'ej M

;DalGwt lgsfodf cg'ej M

!= s] tkfO{n] o; ;+:yfn] u/]sf jt{dfg sfo{s|dx?sf] af/]df] hfgsf/L lbg ;Sg'x'G5 <

@= o; ;+:yfdf l6=aL=/ Pr=cfO=eL= lgoGq0fgsf nflu s] s:tf lsl;dsf sfo{s|dx? ;+rfng eO/x]sf 5g\ <

#= o; ;+:yfn] Ifo/f]u ;dofdg} lgbfg ug{sf] nflu g]kfn Ifo/f]u sfo{s|dnfO{ s;l/ ;xof]u ul//]x]sf] 5 / ;dodfg} l6=aL= lgbfg ug{sf] nflu s] s:tf of]hgfx? ag]sf 5g\ <

$= Ifo/f]u lgbfg ug{sf] nflu lhg PS;k6{ k|of]u ubf{ tkfO{n] ;a}eGbf a9L hf]lvddf s'g ;d'x kfpFg'eof] <

%= g]kfndf lhg PS;k6{åf/f kl/If0f ug{] sfo{ sfof{Gjog ug{sf] nflu b]lVfPsf d'Vo ;d:ofx? s] s] x'g\ <

^= tkfO{sf] ljrf/df pRr hf]lvd ;d"xx? lhg PS;k6{ ljlwåf/f kl/If0f ug{] sfo{s|ddf ;xeflu ePsf 5g\ <

&= tkfO{sf] ljrf/df lhg PS;k6{ ljlwsf] k|of]u u/]/ s;l/ Ifo/f]u kQf nufpg] (case detection) b/ a9fpg ;lsG5 <

*= tkfO{sf] larf/df lhg PS;k6{ ljlwåf/f Ifo/f]usf] lgoldt lgbfg ug{sf nflu g]kfn Ifo/f]u sfo{s|dn] s:tf of]hgfx? ckgfpg' k5{ <

**wGojfb**

**;/f]sf/jfnfx?;Fu cGt/jftf{ - LAB**

ldlt M

sf]8 g+M

cGt/jf{tf lng] JolQmsf] kl/ro

gfd M

lnË M

kb M

lhNnf M

xfnsf] ;+:yfdf cg'ej M

;DalGwt lgsfodf cg'ej M

!= s] tkfO{n] o; If]qsf] l6=aL= / Pr=cfO=eL= sf] jt{dfg cj:yfaf/]df hfgsf/L lbg ;Sg'x'G5<

@= o; ;+:yfdf l6=aL= lgbfgsf nflu s] s:tf lsl;dsf sfo{s|dsf ;+rfng eO/x]sf 5g\ <

#= o; ;+:yfaf6 ;dofdfg} l6=aL=lgbfg ug{sf] nflu tkfOn] s] s:tf of]hgfx? agfpg'ePsf] 5 <

$= l6=aL=lgbfg ug{sf] nflu lhg PS;k6{ k|of]u ubf{sf] tkfO{sf] cg'ej s:tf] /x\of] <

%= l6=aL=lgbfg ug{sf] nflu s'g kl/If0f ljlw a9L ;lhnf] dfGg'x'G5 < -lhg PS;k6{ jf dfOs|f]:sf]kåf/f vsf/ kl/If0f_

^= lhg PS;k6{åf/f kl/If0f ug{] sfo{ sfof{Gjog ug{sf] nflu b]lVfPsf d'Vo ;d:ofx? s] s] x'g\<

-ljB't cfk"lt{, cartridge sf] cfk"lt{ /fli6«o Ifo/f]u s]Gb|åf/f dd{tsf] ;'ljwf_

&= hf]lvd ;d"xx? h:t} Pr=cfO=eL= ;ª|s|ldt JolQm, afnaflnsf cflb tkfO{sf] lhg PS;k6{ ljlwåf/f tkfO{sf] k|of]uzfnfdf kl/If0f ug{ OR5's 5g\ <

*= tkfO{sf] ljrf/df lhg PS;k6{ ljlwsf] k|of]u u/]/ s;l/ Ifo/f]u kQf nufpg] b/ a9fpg ;lsG5 <

(= cTodf lhg PS;k6{sf] lkmtnf] sfof{Gjogsf] ;d:of ;'Nemfpg / lgbfgsf] glag pks/0f sf] k|of]u u/]/ Ifo/f]uLsf]] ;+Vof a9fpg s] ;'emfj lbg rfxg'x'G5 <

**wGojfb**

**;/f]sf/jfnfx?;Fu cGt/jftf{ - NTP**

ldlt M

sf]8 g+M

cGt/jf{tf lng] JolQmsf] kl/ro

gfd M

lnË M

kb M

lhNnf M

xfnsf] ;+:yfdf cg'ej M

;DalGwt lgsfodf cg'ej M

!= g]kfndf Ifo/f]usf] jt{dfg cj:yf s:tf] 5 <

@= o; ;+:yfaf6 l6=aL= tyf Pr=cfO=eL=lgoGqsf nflu s] s:tf lsl;dsf sfo{s|dsf ;+rfng eO/x]sf 5g\ <

#= o; ;+:yfaf6 ;dofdfg} l6=aL=lgbfg ug{sf] nflu tkfOn] s] s:tf of]hgfx? agfpg'ePsf] 5 <

$= l6=aL=lgbfg ug{sf] nflu lhg PS;k6{ k|of]u ubf{sf] tkfO{sf] cg'ej s:tf] /x\of] <

%= g]kfndf Ifo/f]usf hf]lvd ;d"xx? s'g s'g x'g\ <

^= lhg PS;k6{åf/f kl/If0f ug{] sfo{ sfof{Gjog ug{sf] nflu b]lVfPsf d'Vo ;d:ofx? s] s] x'g\<

-ljB't cfk"lt{, cartridge sf] cfk"lt{ /fli6«o Ifo/f]u s]Gb|åf/f dd{tsf] ;'ljwf_

&= hf]lvd ;d"xx? h:t} Pr=cfO=eL= ;ª|s|ldt JolQm, afnaflnsf cflb tkfO{sf] lhg PS;k6{ ljlwåf/f tkfO{sf] k|of]uzfnfdf kl/If0f ug{ OR5's 5g\ <

*= tkfO{sf] ljrf/df lhg PS;k6{ ljlwsf] k|of]u u/]/ s;l/ Ifo/f]u kQf nufpg] ;+Vof a9fpg ;lsG5 <

(= cTodf lhg PS;k6{åf/f kl/If0f ug{] ljlw :s]n ck ug{ s]lx of]hgfx? ag]sf 5g\ <

**wGojfb**

**;/f]sf/jfnfx?;Fu cGt/jftf{ - WHO**

ldlt M

sf]8 g+M

cGt/jf{tf lng] JolQmsf] kl/ro

gfd M

lnË M

kb M

lhNnf M

xfnsf] ;+:yfdf cg'ej M

;DalGwt lgsfodf cg'ej M

!= s] tkfO{n] g]kfndf Ifo/f]u / Pr=cfO=eL=sf] jt{dfg cj:yf s:tf] 5 eGg] af/]df ;+lIfKt hfgsf/L lbg ;Sg'x'G5 <

@= o; ;+:yfdf l6=aL= lgbfgsf nflu s] s:tf lsl;dsf sfo{s|dsf ;+rfng eO/x]sf 5g\ <

#= ljZj :Jff:Yo ;+u7gn] ;dofdfg} l6=aL=lgbfg ug{sf] nflu s] s:tf of]hgfx? agfPsf] 5 <

$= l6=aL=lgbfg ug{sf] nflu lhg PS;k6{ k|of]u ubf{sf] tkfO{sf] cg'ej s:tf] /x\of] <

%= l6=aL=lgbfg ug{sf] nflu s'g kl/If0f ljlw a9L ;lhnf] dfGg'x'G5 < -lhg PS;k6{ jf dfOs|f]:sf]kåf/f vsf/ kl/If0f_

^= lhg PS;k6{åf/f kl/If0f ug{] sfo{ sfof{Gjog ug{sf] nflu g]kfndf b]lVfPsf d'Vo ;d:ofx? s] s] x'g\<

-ljB't cfk"lt{, cartridge sf] cfk"lt{ /fli6«o Ifo/f]u s]Gb|åf/f dd{tsf] ;'ljwf_

&= hf]lvd ;d"xx? h:t} Pr=cfO=eL= ;ª|s|ldt JolQm, afnaflnsf cflb tkfO{sf] lhg PS;k6{ ljlwåf/f lgbfg eO/x]sf 5g\ <

*= cTodf lhg PS;k6{sf] lkmtnf] sfof{Gjogsf] ;d:of ;'Nemfpg / lgbfgsf] glag pks/0f sf] k|of]u u/]/ Ifo/f]uLsf]] ;+Vof a9fpg s] ;'emfj lbg rfxg'x'G5 <

**wGojfb**

**d~h'/Lgfdf**

**ldlt================================**

**sf]8 g+== ==========================**

gd:sf/ d]/f] gfd a;Gt hf]zL xf] . d clxn] hg:jf:Yo ;+sfo cGtu{t ufhfdfbf ljZjljBfno OG8f]g]l;ofdf :gfts]fQ/ txdf cWoog/t 5' . oxL l;nl;nfdf **“g]kfndf Ifo/f]u lgbfgsf] nflu lhg PS;k6{ k|ljlw sfof{Gjog”** zf]wkq tof/ ug{sf nflu of] cWoog ub}{5' . ;f] cWoogsf] l;nl;nfdf tkfO{n] lbPsf ;"rgfx? Uff]Ko /flvg] 5g\ / cWoog p¢]Zosf nflu dfq k|of]u ul/g]5g\ . olb tkfO{ o; cWoogdf cfˆgf] wf/0ff lbgsf] nfuL d~h'/ x'g'x'G5 eg] d tkfO{;Fu k|Zg lng cg'dlt rfxG5' .

!= d~h'/ 5'

@ d~h'/ 5}g

cGt/jftf{ lbg] JolQmsf] b:tvt =============

cGt/jftf{ lng] JolQmsf] gfd ======================

cGt/jftf{ lng] JolQmsf] b:tvt =======================
